# Ensuring Optimal Mental Health Programs and Policies for First Responders: Opportunities and Challenges in One U.S. State

**DOI:** 10.1007/s10597-023-01121-1

**Published:** 2023-03-22

**Authors:** Michael P. Fisher, Catherine D. Lavender

**Affiliations:** 1 Department of Health Policy, Management & Leadership, School of Public Health, West Virginia University, P.O. Box 9190, Morgantown, WV 26505 USA; 2 Department of Occupational Therapy & Occupational Science, College of Health Professions, Towson University, 8000 York Road, Linthicum Hall, Towson, MD 21252 USA

**Keywords:** Mental health policy, First responder, Peer support, Critical incident stress management, Stigma, Suicide

## Abstract

This study examined opportunities and challenges faced by individuals working to advance mental health policy and programming for first responders. We utilized qualitative content analysis and interviews with 16 firefighters, emergency medical services professionals, law enforcement officers, and others involved in programming or policy in the U.S. state of Ohio. Six themes characterized opportunities and challenges encountered: (1) variations in programming and policy exist across jurisdictions; (2) opportunities exist to enhance mental health awareness and self-care training for first responders; (3) need exists for specialized mental health clinicians accustomed to and capable of effectively working with first responders; (4) confidentiality protections are lacking for peer supporters not trained in critical incident stress management; (5) having an internal champion and broader support is key to program and policy advancement; and (6) interdepartmental collaboration provides opportunities for sharing resources and best practices. Results illustrate continued need for strategic policymaking, program development, and coordination.

## Introduction

Psychological impacts such as anxiety, depression, posttraumatic stress disorder (PTSD), sleep disorders, substance misuse, elevated suicide risk, and family or relationship issues have long been associated with high-stress occupations, including some first responder professions (Berger et al., 2012; Boffa et al., 2017; Casas & Benuto, 2022; Jetelina et al., 2020; Jones et al., 2018; Lewis-Schroeder et al., 2018; McFarlane et al., 2009). Events such as the COVID-19 pandemic, the opioid epidemic, and mass casualties may intensify stressors among first responders. Firefighters, law enforcement officers, emergency medical services (EMS) professionals, and others may face longer hours, added responsibilities, and elevated exposure to potentially traumatic events (Ehrlich et al., 2020; Hoffman, 2020; Jozaghi et al., 2018; Klimley et al., 2018; National Institute for Occupational Safety and Health, 2021; Pike et al., 2019; Stogner et al., 2020). Meanwhile, many first responder communities are bolstering their efforts to develop, implement, and improve upon programs and policies supporting mental health and well-being.

Across jurisdictions, first responders can often receive mental health services, when needed, through several channels. One such avenue is employee assistance programs (EAPs), work-based initiatives offering wide-ranging services—including mental health treatment and referral—for individuals with personal or work-related concerns (Office of Personnel Management, n.d.). In addition, first responders with adequate health insurance coverage or ability to pay may seek care via mental health providers in the community (e.g., private practitioners), some of whom focus on first responder populations (Gulliver et al., 2019; Jones et al., 2020). Aside from treatment, per se, first responders may also participate in critical incident stress debriefing (CISD) or peer support activities, where available. CISD is a standardized activity used following a traumatic incident which seeks to educate about stress and coping, normalize stress reactions, promote emotional processing, and provide referral information (Klimley et al., 2018; Litz et al., 2002). It is one component of an umbrella approach to crisis intervention known as critical incident stress management (CISM). Peer support programs train selected first responders to provide social and emotional support to peers in distress due to occupational or personal factors (Klimley et al., 2018; Jones et al., 2020). Both programmatic activities are widely (though not universally) available and can facilitate early intervention and referral to treatment. In addition, chaplain programs and spouse support programs sometimes augment or work in conjunction with first responder mental health or well-being programs (National Alliance on Mental Illness, 2023; Ramchand et al., 2019).

Public and organizational policies have also been developed or expanded to address the mental health and well-being of first responders. For example, the State of Ohio passed legislation in 2021 to examine expanding eligibility for workers’ compensation benefits for first responders suffering from PTSD (H.B. 308, 2021). In addition, Ohio implemented a state-level office focused on the well-being of first responders to coordinate specialized support and training (Ohio.gov, 2021). Local policies have also emerged. For example, the city of Cleveland and local union representatives negotiated a collective bargaining agreement in 2019 enabling emergency medical technicians (EMTs), paramedics, and dispatchers to request and receive at least one hour off duty to decompress after potentially traumatic events (Ohio State Employment Relations Board, 2019). At the federal level, members of Congress introduced legislation in 2022 (S.4007) and 2023 (H.R.472) aimed at establishing a program making state-of-the-art treatment and preventive care for PTSD or acute stress disorder widely available to first responders (Fighting Post-Traumatic Stress Disorder Act, 2022; Fighting Post-Traumatic Stress Disorder Act, 2023). Moreover, increased federal attention has been placed on the issue of first responder suicide (e.g., Tiesman et al., 2021; Helping Emergency Responders Overcome Act, 2021).

Despite proliferation of mental health programs and policies for first responders, literature on the implementation and overall extent of these efforts is limited (Spence et al., 2019). At least a handful of studies have assessed factors that help or hinder mental health program implementation (e.g., Knaak et al., 2019; McKeon et al., 2021). Studies have also examined related topics such as barriers to obtaining mental health services (e.g., Haugen et al., 2017; Jones et al., 2020) and the effectiveness of mental health programs (e.g., Thompson & Drew, 2020; Boothroyd et al., 2019). However, most such studies tend to focus on a specific program—often its outcomes—rather than the larger landscape of programs and policies. Accordingly, little is known about factors promoting or inhibiting mental health program or policy advancement as well as the existence (or absence) of programs and policies across jurisdictions. Without adequate research in these areas, policymakers and organizational leaders may lack insights crucial to program or policy development or implementation. Thus, the goal of this exploratory research is to assess how first responders involved in mental health program or policy activities in one U.S. state (Ohio) are responding to mental health issues.

## Methods

This research was guided by content analysis methodology (Hsieh & Shannon, 2005), a systematic approach capable of providing an in-depth understanding of the topic. As is typical of qualitative research, our study was guided by research questions rather than hypotheses. Specifically, we assessed (1) how Ohio first responder communities are advancing mental health programs and policies and (2) the opportunities and challenges faced. The state of Ohio was chosen because notable mental health program and policy changes have been enacted recently.

Data were collected between March 2021 and June 2022. We conducted semi-structured 60-minute interviews via videoconference (Zoom) or telephone with individuals in Ohio involved in first responder mental health programming or policy. Fifty-six individuals were contacted for participation using a standardized email script with study description, and 16 participated. This resulted in a response rate of 28.6%. Verbal informed consent was obtained prior to interviews. Three first responder occupations—firefighters, EMS professionals, and law enforcement officers—were the focus of the study. This ensured broad representation since policies pertaining to first responder mental health often address these professions. Of participants, 4 (25%) were employed in larger cities (> 250k), 8 (50%) in medium-sized cities (25k-250k residents), and 3 (19%) in smaller townships or villages (< 25k residents) (U.S. Census Bureau, 2019). One participant (6%) was employed by a state-level organization.

Participants were purposively sampled based on their roles within organizations, with priority given to those serving as first responders but also involved in mental health programming or policy. This included individuals who had advocated for mental health programs or policies, served as peer supporters, or occupied positions on boards or committees addressing first responder well-being. No further inclusion or exclusion criteria were applied. Snowball sampling was also used. Interviews continued until we reached thematic saturation, the point at which no new properties of relevant themes emerged (Charmaz, 2014).

Interviews were conducted by the principal investigator, a male doctoral-trained mixed methods researcher with no prior relationships with participants. Likewise, study participants had little knowledge of the interviewer aside from profession (assistant professor) and research interest (first responder mental health policy). Interview topics corresponded to the study aims described above. An overarching goal during interviews was to elicit data pertaining to multiple dimensions of program and policy development or implementation, ranging from personal attitudes, beliefs, and behaviors to broader political and organizational practices. Both programs and policies were topics during interviews since the two are often interrelated and some first responders were involved in both. For example, certain mental health program staff were involved in the creation of a program but had also advocated in support of a policy.

Interviews were audio-recorded, professionally transcribed, and managed in Dedoose version 9.0.46. Interview data were analyzed in accordance with inductive or “conventional” qualitative content analysis methodology (Hsieh & Shannon, 2005). Transcribed data were coded using a scheme of approximately 60 codes or labels derived from the data and pertaining to various aspects of program or policy development or implementation. Ongoing coding procedures enabled the elaboration of codes and the clustering of related codes into categories and themes. Themes were dichotomized into two major categories: (1) a changing professional context (comprising three themes) and (2) opportunities and challenges (comprising six themes). Transcripts were coded by one researcher, though 25% of transcripts were also coded independently by the other researcher to ensure the validity of codes and consistency of their application (Morse, 2015). The researchers met regularly to discuss coding and resolve any coding discrepancies through consensus. Hand-written field notes were taken during interviews and used to augment or clarify topics discussed. Memoing was utilized throughout the research as a reflexive and analytical tool. Data reporting is consistent with COREQ guidelines (Tong et al., 2007). To ensure participants had an opportunity to validate the accuracy and completeness of the findings, a participant checking strategy was utilized (Creswell & Miller, 2000). Specifically, a summary of thematic content was presented to three-fourths of participants (12 of 16), and feedback was received from five of those individuals and then incorporated.

The study was approved by the West Virginia University Institutional Review Board (No. 2208638968) and the Towson University Institutional Review Board (No. 1381). The authors have no competing interests to declare that are relevant to the content of this article. All authors certify responsibility for the manuscript.

## Results

Sixteen individuals across 15 departments from 14 jurisdictions completed an interview. Eleven were firefighters, paramedics, or EMS professionals, many with a dual role such as firefighter-paramedic; four were affiliated with law enforcement; and one was a government employee (non-first responder). Fourteen were male, and two were female. Interviewees had been in their general profession for at least five years (range: 5–37 years). Education levels varied and included some college (n = 4), associate degree (n = 2), bachelor’s degree (n = 5), and master’s degree (n = 5). Interviewees shared perspectives on contextual issues surrounding mental health programming or policy and on opportunities and challenges encountered while working in this environment. Below, we describe emergent themes within these two categories.

### A Changing Context

First responders framed mental health program and policy advancements amid a changing professional context, wherein (1) increased professional demands and cumulative stressors are intensifying mental health need; (2) decreases in stigma are enabling mental health awareness and understanding; and (3) first responder suicides are catalyzing mental health programs and policies. These themes are described below.

#### Witnessing Intensified Professional Demands and Cumulative Stressors

Most first responders witnessed recent workload increases in their departments, often in conjunction with staffing constraints. As one firefighter-paramedic expressed: “Our staffing model is stagnant, so [it’s the] same amount of people doing more work. So, I think on that end, we’re seeing more pressure.” Some interviewees emphasized that increased workloads can negatively impact first responder wellness. For example: “If you give me one less person, we’re going to have to give you 130% today in order to make up for it. And we do… we get the job done. But it comes at a cost, right? Emotionally, physically, mentally.” Heightened workloads were particularly salient among EMTs and paramedics but also an issue for other first responder professions.

Certain work and workload challenges were viewed as resultant of the COVID-19 pandemic, given heightened demands and professional uncertainties for some first responders. For example, one firefighter-paramedic shared: “It just scared the shit out of all of us… We didn’t feel like we had adequate PPE. All the policies… were absent, or there were updates [constantly], which made it hard to track… The pure magnitude of it initially was overwhelming.” Others noted the pandemic necessitated hypervigilance about one’s actions and risks. Many first responders were concerned they might contract COVID at work and transmit it to their families. As one paramedic shared:

These guys were going from nursing home to nursing home where everybody had COVID. And they didn’t know if they were going to get it and take it home to their family. It definitely impacted. I mean, I often wonder if I brought it home to my family.

Some felt that personal and professional stressors compounded during the pandemic to create an especially challenging time.

Beyond demands related to COVID-19, many first responders had witnessed broader trends pertaining to work and workload. Some interviewees—particularly those with many years of professional experience—emphasized that today’s work environment is fundamentally more stressful than in decades past, not simply due to increased work volume but also the intensity of situations faced. Notably, increases in violent crime were reported to intensify work-related stress. For example, one paramedic stated: “Violent crime is increasing, and accidents and stuff, so more of what we see is becoming more and more intense.” One firefighter-paramedic explained how a mass casualty event had placed significant pressure on their department’s staff, while another described how a local fire department had acquired ballistic vests and helmets because of an increased threat of violence. Many interviewees highlighted the burden placed on their department by the opioid epidemic. For example, one firefighter-paramedic noted: “Our run volumes are getting larger… The opioid epidemic crushed us, crushed the fire service. Just run after run, and we would have some days upward of 15 overdoses.” Importantly, one firefighter-paramedic emphasized how these various pressures can lead to compassion fatigue: “Between the overdoses and the violence and the shootings… we’re going to several of them every day… we kind of shed it as, ‘this is life.’ But you… get cold to life, right? You get a lot of compassion fatigue.”

Some interviewees noted an added layer of stress or confusion because of negative public perceptions of their profession. This was expressed primarily by interviewees affiliated with law enforcement. For example, one police officer stated: “I think that the big change, which does have impact on mental health, is the whole new media scrutiny, the negativity towards police.” Of note, a similar sentiment was expressed by a paramedic who reported sometimes being mistaken for or characterized alongside law enforcement: “They see us in our uniform with our radio. They look at us with a badge on our shirt. And they say, ‘Oh, you’re a cop.’ ‘No, I’m not. I’m a paramedic. I’m here to help you.’”

#### Experiencing Decreases in Stigma, though Challenges Remain

Nearly all interviewees cited mental illness stigma as a concern for first responder professions but perceived lower stigma levels than in decades past. As one firefighter-paramedic suggested:

The last… 5 to 10 years is when the stigma started to get eroded a little bit more. There was more of a recognition that we have real issues, that PTSD was out there, and [we] had understandings and new understandings of PTSD, of cumulative stress, of cumulative events… we started to recognize compassion fatigue and how that’s affecting our members.

Interviewees viewed the current environment as more accepting of first responders talking openly about mental health topics, disclosing mental health issues to peers, or seeking mental health treatment. Some cited examples of first responders sharing their personal mental health stories. About half of interviewees expressed that younger staff (loosely construed as people in their 20s or 30s) were less likely to stigmatize mental health issues than older staff. For example, one firefighter stated: “We have a younger department, so they’re more open to mental health, because they’ve kind of grown up with it in the schools and just hearing about it.” Alongside reported decreases in stigma, many interviewees also witnessed recent proliferations of programs and services to support first responder mental health.

Despite stated advancements in mental health stigma reduction, some stigma was still reported. For example, one firefighter-paramedic described a cohort of first responders who were hesitant to support others with mental health issues: “I see a divide where there’s some folks that are really on board… which is a lot of people and our administration, but there’s a core group that views first responder mental health as maybe, ‘Well, we’re making people soft.’” Interviewees also noticed that first responders often fear job-related repercussions and therefore do not present with mental health issues. For example, one police officer noted how such fears are manifesting within a police department’s peer support program:

I also have friends… who said that nobody’s talking to [the peer supporters] because… the feeling is that those people are the ones who want to be promoted, and the ones that are looking to get the scoop on you or get some kind of dirt on you so they can use it against you at promotion time or something, coming forward with a mental health issue.

Other interviewees noted that first responders are sometimes reluctant to seek treatment via programs such as EAPs because of stigma concerns or fears that confidentiality could be breached.

#### Observing First Responder Suicide as a Catalyst to Action

Most interviewees viewed first responder suicide as a critical issue within their department or among their peers. Many had experienced the suicide of a colleague (or several colleagues) in recent years. For example, one firefighter-paramedic stated: “[Our department] hasn’t had a line-of-duty death on a fire ground [for decades]. And we’re losing a member a year to suicide… Yeah, so I’ve been here a year and a half, and… I’ve seen two suicides.” Similarly, another firefighter-paramedic noted: “I’m up to 18 of my coworkers over the past 30 to 35 years that have taken their own lives. And that doesn’t mean I was working with them at the time, but I had worked with them over the decades.”

For some, suicide events were seen as “teachable moments” or catalysts to mental health programs, policies, or awareness. In fact, many interviewees noted that first responder suicide was the most salient catalyst of mental health initiatives within their department or jurisdiction. For example, one firefighter-paramedic shared: “I wouldn’t say it on a positive note, but what’s made this all come to life and happen are losses… suicides. In [our department], it took the last suicide that we had, which was probably a year and a half ago.” More broadly, first responders perceived increases in the incidence of suicide and noted that overall rates, aside from specific suicide events, have also prompted awareness. As one law enforcement officer shared: “When suicide among first responders out numbers line-of-duty deaths, it starts to get their attention. I mean, that’s been a big factor.”

### Opportunities and Challenges

Six emergent themes characterized opportunities and challenges in mental health programming or policy: (1) variations in programming and policy exist across jurisdictions, with small or rural departments sometimes facing barriers to robust programming; (2) opportunities exist to enhance mental health awareness and self-care training for first responders; (3) a need exists for specialized mental health clinicians accustomed to and capable of effectively working with first responders; (4) confidentiality protections are lacking for peer supporters not trained in CISM; (5) having an internal champion and broader community or political support is key to mental health program and policy advancement; and (6) interdepartmental collaboration provides opportunities for sharing resources and best practices (see Table 1). These themes are described below.

**Table 1:** Summary of opportunities and challenges in mental health programming or policy

Theme	Description
1. **Variations in programming and policy** exist across jurisdictions, with small or rural departments sometimes facing barriers to robust programming.	a. Certain programs (peer support, CISM, employee assistance programs) are common across departments, but others are unique or novel. b. Health insurance benefits can vary and may be lacking among volunteer or part-time employees. c. Overall, variation is viewed both positively and negatively, depending on the circumstances.
2. Opportunities exist to enhance **mental health awareness and self-care training** for first responders.	a. First responders receive little or no training in mental health awareness and self-care, with training type and duration differing by profession. b. Additional training is seen as desirable.
3. A need exists for **specialized mental health clinicians** accustomed to and capable of effectively working with first responders.	a. There exists a need for clinicians who are knowledgeable about and culturally sensitive to issues first responders face. b. Some departments or programs engage in their own efforts to address the issue.
4. **Confidentiality protections** are lacking for peer supporters not trained in Critical Incident Stress Management (CISM).	a. Peer supporters who are trained in CISM are protected under confidentiality laws covering any peer discussions extending from CISM activities. b. Peer supporters lacking CISM training are not protected under those confidentiality laws.
5. Having an **internal champion and broader community or political support** is key to mental health program and policy advancement.	a. Robust mental health activities are often the result of grassroots efforts led by a champion rather than the consequence of top-down directives. b. Champions are often aided by broader community or political support.
6. **Interdepartmental collaboration** provides opportunities for sharing resources and best practices.	a. Some programs, especially peer support programs, collaborate with other departments or jurisdictions. b. Some peer support organizations share best practices with other programs or benefit from information shared with them.

#### Variations in Programming and Policy Exist Across Jurisdictions, with Small or Rural Departments Sometimes Facing Barriers to Robust Programming

Interviewees described differing program and policy efforts, with large jurisdictions reporting more expansive mental health resources and infrastructure. For example, several municipalities and especially those in large urban areas had extensive peer support programming, while capacities in other areas appeared less robust. While programs such as peer support, CISM, and EAPs appeared common across departments, other programs were more unique or novel. For example, one large jurisdiction operates an internal EAP (whereas most rely on external EAPs). Some departments utilize mental health-focused apps to connect first responders to resources. Others have regular mental health checks or provide direct access to clinicians who specialize in first responder mental health. Unique or novel policies also exist. For example, one large municipality has a collective bargaining agreement in place with stipulations concerning first responder mental health. Meanwhile, some departments informally permit first responders to use personal leave, if needed, immediately following a significant stressor (e.g., by going home for the afternoon).

Variation reportedly manifests in health insurance benefits also. For example, one firefighter-paramedic in a large metropolitan area suggested that well-funded jurisdictions offer insurance plans with better mental health coverage than locales facing significant resource constraints: “In a large department, we’re much better at taking care of our people. We have much better resources and much better benefits.” Other interviewees reported that volunteer or part-time firefighters in rural areas often lack health insurance or sufficient mental health coverage and therefore have limited treatment options. For example, one firefighter-paramedic stated: “We have a lot of volunteer and part-time departments around us… And they call needing help, but they don’t have health insurance. And finding them help without health insurance, that is our biggest hurdle.”

Importantly, variation was viewed both positively and negatively. Certain unique programs and policies were seen as beneficial to first responder mental health and were highly valued. At the same time, interviewees were skeptical of programs whose evidence had not been established. Indeed, the proliferation of non-evidence-based programs can reportedly create difficulties for first responders trying to obtain effective care. As one firefighter-paramedic observed:

There’s a lot of bandwagons out there. And we’ve got some people that might be trying to promote their own program. Or everybody seems to want to be involved, but they’re playing under different rules in different areas. Just the coordination thing, I think, is huge. When you have somebody that’s in a crisis, they don’t have the capacity to go through a list of 100 people.

Some variation in programming and policy was not viewed as especially positive or negative but was instead seen as a product of the local environment. For example, an interviewee at a rural fire department explained that their firefighters faced less intense situations than many other departments and therefore did not require the same level of mental health resources.

#### Opportunities Exist to Enhance Mental Health Awareness and Self-Care Training for First Responders

Many interviewees highlighted that first responders receive little or no training in mental health awareness and self-care, with training type and duration differing by profession. For example, one firefighter-paramedic noted: “On the fire side and EMS side, mental health is not talked about. I just went through a paramedic refresher through a third-party agency… But there was no discussion about my mental health.” While such trainings, when in place, were perceived as helpful, they were not necessarily seen as sufficient. Many desired additional training activities upon initial certification or later via continuing education. For example, one firefighter-paramedic expressed:

I wish there was more—if the state curriculum dictated either ‘X’ number of hours or a curriculum for mental health, I think that would be really helpful... And if it was normalized through curriculum from the state, just like training for EMS, training for fire, I think that would be a game-changer.

Some were actively working to provide additional mental health awareness training within their department via, for example, ad-hoc sessions led by peer supporters.

#### A Need Exists for Specialized Mental Health Clinicians Accustomed to and Capable of Effectively Working with First Responders

Several interviewees highlighted challenges that first responders may encounter when seeking treatment from clinicians who are not knowledgeable about or culturally sensitive to issues first responders face. Such clinical encounters were described as useless or potentially harmful to first responders. For example, one firefighter-paramedic stated:

They don’t necessarily get us. There’s a lot of stories out there where the firefighter goes to the clinician and by the end of the session the clinician’s the one crying on the couch… We’re dealing with the worst of the worst: the worst sights, sounds, smells, and everything.

Reportedly, some clinicians and treatment centers have marketed themselves to first responders when in fact they lack specialized expertise. For example, one firefighter-paramedic commented: “There are a lot of folks in Ohio who market to us, but it’s clever marketing, and then we get thrown in with the general population… That’s not acceptable.” Some interviewees viewed referrals from EAPs and health insurers as likely avenues for encountering clinicians not culturally competent in first responder mental health.

Addressing this need for culturally competent clinicians, one jurisdiction maintained an in-house EAP that refers first responders to clinicians who have been properly vetted. A handful of other jurisdictions arranged, either formally or informally, for an appropriately trained clinician (or group of clinicians) to be available to their employees. Some interviewees mentioned their own department’s efforts to educate clinicians on first responder culture through “ride-alongs” that exposed clinicians to daily life experiences of the profession.

#### Confidentiality Protections are Lacking for Peer Supporters Not Trained in Critical Incident Stress Management

Several interviewees noted that peer supporters who are trained in CISM by the International Critical Incident Stress Foundation (ICISF) are protected under confidentiality laws and cannot be mandated to disclose details of peer discussions extending from CISM activities. However, interviewees expressed concern that peer supporters lacking this training would not be protected under those confidentiality laws. As one firefighter-paramedic summarized:

We don’t have legal protection when it comes to communication between peer support members. So, for example, if a police officer was involved in using deadly force, right, and they were distraught about it, and they went to a peer supporter and said, ‘I’m distraught about the use of deadly force. Here is why.’ [If] that police officer gets caught up in litigation, gets called into court, that peer supporter right now would be mandated to provide testimony on what was divulged in that peer support session.

Some interviewees suggested their department’s peer supporters had been trained in CISM, while others reported their peer supporters had not. Overall, confidentiality protections for peer supporters were a high priority and concern for nearly all interviewees.

#### Having an Internal Champion and Broader Community or Political Support is Key to Mental Health Program and Policy Advancement

Most departments with wide-reaching mental health programs or policies had one or more champions who spearheaded efforts. In other words, robust mental health activities were often the result of grassroots efforts within or across departments rather than top-down directives. Examples cited during interviews included first responders who worked to create a peer support team within their department or served in a leadership role and promoted mental health during their tenure. For example, one firefighter-paramedic peer supporter shared:

It was really grassroots. A couple of us who have suffered throughout our careers with different issues... went to the peer support through the [International Association of Fire Fighters] program. And we just started shooting the crap around a little bit. And next thing I know, we’ve got this program.

Internal champions were often aided by broader community or political support. For example, interviewees expressed support from city leaders, state congressional members, and other figures in the community. Implicit in such situations was the support of internal leaders such as police or fire chiefs.

#### Interdepartmental Collaboration Provides Opportunities for Sharing Resources and Best Practices

Several interviewees reported benefitting from collaboration with other departments or jurisdictions. This was especially evident among peer support programs. Some programs had been created explicitly at the county or regional level rather than city or departmental levels, thus facilitating wide availability of peer support resources across jurisdictions. But aside from these collaborative peer support organizations, some programs maintained less formal resource sharing arrangements across departments or jurisdictions; these were described by some as “mutual aid agreements for mental health.” As one firefighter-paramedic summarized:

When we set this [peer support program] up, we realized that not every department can afford to have a peer supporter go through the training… They don’t have the budget. So, we will go to them… to establish that relationship. ‘You guys need something, you can call me.’ It’s sort of like mutual aid for mental health.

Indeed, several peer supporters we interviewed reported often making themselves available to other departments.

In addition to sharing resources, some peer support organizations reported sharing information and best practices with other programs. For example, one firefighter-paramedic noted: “It started with [our] peer support team… as we were putting this together… when it came to writing policy, I basically just adopted—we went out and found some other people’s policies that existed.” Interviewees who reported borrowing mental health programming strategies from other locales did so, at least in part, to avoid “recreating the wheel” and reported benefitting from the shared information.

## Discussion

This study illuminates opportunities and challenges faced by first responders involved in mental health programming or policy in one U.S. state and describes the changing professional context in which these individuals are working. First responders have faced several opportunities and challenges, ranging from a need for specialized mental health clinicians accustomed to and capable of effectively working with first responders to variation in programming or policy across jurisdictions. Additionally, our results indicate that some first responders are operating amid a changing professional context wherein, for example, increased professional demands and cumulative stressors are intensifying mental health need. Our results have several implications for first responder mental health policy and planning. Below, we discuss some of the more pressing priorities and offer five recommendations aimed at policymakers and others concerned with first responder mental health. These recommendations are especially relevant within the state of Ohio but appear to complement national research findings and possess broader applicability (Copple et al., 2019; Spence et al., 2019).

### Explore the Utility and Replicability of Strategies for Connecting First Responders with Culturally Competent Clinicians

Our findings indicate a continued challenge in linking first responders with mental health professionals knowledgeable about and culturally sensitive to issues first responders face (Spence et al., 2019; Crowe et al., 2022). Research suggests that first responders often prefer mental health clinicians who are experienced in treating these populations (Spence et al., 2019; Crowe et al., 2022; Jones et al., 2020; Papazoglou & Tuttle, 2018). Accordingly, the U.S. Department of Justice (DOJ) has recommended that law enforcement entities expand specialized provider training and resources and embed qualified mental health professionals into law enforcement agencies (Spence et al., 2019). Similarly, our results suggest that systematic access to culturally competent clinicians is desired across first-responder professions. Some interviewees shared knowledge of various initiatives for linking first responders with culturally competent clinicians. These efforts were typically informal, ad-hoc, and specific to one department or jurisdiction. For example, one firefighter-paramedic described a model wherein a culturally competent provider had been introduced to members of the fire department and was located nearby, though not embedded, per se. These types of unique approaches hold promise, and their utility and replicability should be explored further (Spence et al., 2019).

### Establish (or Assign) One or More Centralized Organizations to Coordinate Policy and Programmatic Activities

Another key result of this study is the variation in programs and policies existing across departments and jurisdictions. Variation manifests, for example, in differential access to mental health programs or services, varied self-care or awareness training initiatives, wide-ranging policies and practices concerning time off following a stressful event, and the presence (or lack) of mental health checks. Some variation is inevitable and perhaps a neutral or positive attribute. Notably, certain departments may encounter fewer traumatic incidents and therefore require fewer mental health resources than other departments (though trauma exposure may not be easily predictable based on the size or rurality of a department; see Chopko et al., 2015). However, other variations could prove problematic—for example, when peer support resources are not easily accessible. Our findings, along with extant research and analysis, suggest it may be useful for centralized organizations—for example, at the state level—to coordinate efforts. Such organizations could be responsible for gathering information on the landscape of programs and policies in existence, assessing program effectiveness, facilitating the dissemination of best practices, implementing training, serving as a hub for information sharing, or addressing key issues relevant to first responder mental health (e.g., gaps in peer supporter confidentiality; see Spence et al., 2019). Professional organizations such as the International Association of Fire Fighters and Fraternal Order of Police have advanced some such efforts, particularly in the realm of education and training (Fraternal Order of Police, 2022; International Association of Fire Fighters, 2022). Moreover, a small number of states, including Ohio, have created offices focused on first responder well-being that may be uniquely positioned to coordinate various efforts (Kearl, 2022; Ohio.gov, 2021).

### Examine Models of Regional Collaboration, and Replicate where Appropriate and Feasible

In addition to or in lieu of centralized efforts, local collaborations are another available mechanism for broadening and standardizing activities across regions. Indeed, experts have recognized the importance of supporting and advancing regional programming, particularly peer support (Spence et al., 2019). Examples of regional peer support collaborations spanning multiple departments or jurisdictions were evident in our sample. These collaborations enable smaller departments without peer supporters to easily access peer resources. Any such models deemed successful may warrant replication in areas where need exists. However, as evidenced through our findings, localized efforts tend to rely on an internal champion and broader political or community support; thus, areas without extensive support or resources might need to designate one or more persons responsible for program development and implementation.

### Expand Mental Health Policy and Program Research and Evaluation Efforts

Research and evaluation are critical for determining how best to advance first responder mental health. Future studies should focus on different states and regions to assess program and policy variation more broadly. This should include evaluation research to assess the effectiveness of unique or novel programs. Additionally, contextual factors should be examined to determine how first responder mental health needs may be changing alongside intensifying professional demands and cumulative stressors. Based on emergent evidence, it is reasonable to hypothesize that societal events such as the COVID-19 pandemic, the opioid epidemic, and mass casualties could place additional stress on first responders, especially if departments are not adequately staffed (Ehrlich et al., 2020; Hoffman, 2020; Jozaghi et al., 2018; Klimley et al., 2018; National Institute for Occupational Safety and Health, 2021; Pike et al., 2019; Stogner et al., 2020).

### Pass Legislation to Advance Mental Health Policy and Program Coordination and Implementation

Prioritization of first responder mental health among federal and state lawmakers can help to advance coordination and implementation efforts. The Law Enforcement Mental Health and Wellness Act of 2017 is one such example. It prompted the DOJ to examine mental health practices and services in the U.S. Departments of Defense (DoD) and Veterans Affairs (VA) that could be adopted by law enforcement agencies (Spence et al., 2019). Indeed, these military-focused organizations aim to coordinate and standardize mental health programs and policies and are often seen as models for first responder communities (Spence et al., 2019; Weinick et al., 2011). While that same level of centralization cannot be expected among first responder professions, given the more than 17,000 law enforcement agencies and 29,000 fire departments in the U.S., some such coordination may be plausible and beneficial (Spence et al., 2019; National Fire Protection Association, 2021). To this end, more recently proposed legislation, the Fighting Post-Traumatic Stress Disorder Act of 2023 (H.R.472), if passed, would direct the DOJ to examine the conditions and resources needed to administer programming ensuring the availability of treatment or preventative care for first responders (e.g., evidence-based trauma-informed care, peer support, or counseling). While many logistical details have yet to be determined, this represents a potential step toward more coordinated and widely accessible mental health programming.

### Strengths and Limitations

Importantly, this study has both strengths and limitations. It is among a handful of works focused on first responder mental health programming and policy both within and across organizations. Therefore, this research offers a unique lens into topics such as the variation existing across departments and jurisdictions. However, this study is limited in a few ways. First, our recruitment resulted in a relatively low response rate (28.6%), and it is not clear whether unique perspectives were inadvertently omitted from our sample. In addition, we focused on certain first responder professions, and it is possible that other first responders (e.g., emergency dispatchers) could have different views. Further, there is limited ability to generalize or “transfer” the results to other populations and contexts since the data reflect a small purposive sample in one U.S. state. Nonetheless, it is plausible that other states are facing similar opportunities and challenges, and a limited body of evidence appears to support this hypothesis (Copple et al., 2019; Spence et al., 2019). Studies with larger sample sizes spanning several states may help to confirm the transferability of these results.

## Conclusion

A continued need exists for effective programs and policies supporting first responder mental health. Federal, state, and local governments and non-governmental stakeholders should prioritize strategic policymaking and program development and implementation. Knowing the various challenges and opportunities encountered during the process can inform planning efforts.
